# Management of Acute Coronary Syndrome in the COVID Era

**DOI:** 10.14797/mdcvj.1049

**Published:** 2021-12-15

**Authors:** Ronak Bahuva, Joe Aoun, Sachin S. Goel

**Affiliations:** 1 University at Buffalo, Buffalo, NY, US; 2 Houston Methodist DeBakey Heart & Vascular Center, Houston, TX, US

**Keywords:** COVID-19, acute coronary syndrome, STEMI, NSTEMI, chest pain

## Abstract

Management of acute coronary syndrome (ACS) has emerged as a challenge during the COVID-19 era. There has been a significant increase in the morbidity and mortality associated with ACS both as a direct and an indirect consequence of the pandemic. In this review, we provide an overview of the impact of COVID-19 on patients presenting with ACS and current practices for managing patients presenting with chest pain during the pandemic and for ensuring safety of healthcare professionals. We also discuss treatment strategies and post-ACS care along with current and future perspectives for management of ACS during future waves of COVID-19 infection or similar pandemics.

## Introduction

The coronavirus 2019 (COVID-19) pandemic has significantly impacted management of cardiovascular emergencies across the world. Early in the pandemic, an unforeseen decline in hospital admissions of patients with acute cardiovascular emergencies was noted.^1^ A survey conducted by the Society for Cardiovascular Angiography and Interventions (SCAI) showed that people considered going to a hospital a high-risk behavior for contracting COVID-19, and people over the age of 60 years were more afraid of contracting the disease than of having a heart attack.^2^ This along with extensive public health campaigns promoting staying at home has, in part, led to fewer patients seeking care for symptoms.^3^ Furthermore, the overall reduction in respiratory infections and vigorous exercise related to social isolation may have contributed to a decrease in the incidence of acute coronary syndrome (ACS). However, a significant number of patients have presented with late-stage cardiac diseases, such as late-presenting ACS, and with serious cardiac complications including cardiac arrests and out-of-hospital death.^4,5^ With improved awareness of the disease and public messaging, subsequent COVID-19 waves have not correlated with a similar decline in acute myocardial infarction-related hospitalizations.^6^ The pandemic has further given rise to an immense demand for resources, leading to diversion and deferral of essential preventive and elective medical services for patients with stable cardiac problems.^7^

Increased morbidity and mortality were noted secondary to ACS and were thought to be related to a significantly longer time from symptom onset to hospital presentation in addition to the delay in performing primary percutaneous coronary interventions (PPCI).^4,8^ Managing late-presenting myocardial infarction (MI) has always been challenging and complex, and COVID-19 has added another layer of complexity. In the US, a statistically significant increase in deaths due to ACS has been recorded during the pandemic, correlating geographically with the number of COVID-19 cases in the area.^7^ Early in the pandemic, prospective data from 55 international centers was used to create a “COVID-ACS” registry that included patients who were either COVID-19 positive or had a high index of clinical suspicion for the infection. Analysis of registry data showed that mortality and complications were significantly higher in COVID-ACS patients compared to the same population in the pre-pandemic era (COVID–ST-elevation myocardial infarction [STEMI]: 23% vs 6%, *P* < .001; COVID–non-ST-elevation ACS: 7% vs 1%, *P* < .001; cardiogenic shock in COVID-STEMI: 20% vs 9%*, P* < .001).^9^ For this reason, it is important to address ACS in patients with COVID-19 differently to improve morbidity and mortality while protecting the treatment staff.

## Presentation and Diagnosis

Effectively triaging patients presenting with cardiopulmonary symptoms has become challenging during the COVID-19 pandemic. Healthcare workers in cardiac catheterization laboratories managing patients presumed to be infected with COVID-19 have higher risks of infection than those managing patients with low risk of infection (2.3% vs < 0.25%).^10^ Thus, any patient presenting to the emergency department or clinic should be considered as possibly infected with COVID-19, and a surgical mask should be used as a bare minimum layer of protection when examining the patient.^11,12^ Patients with a high possibility of or known COVID-19 infection should be examined using adequate personal protective equipment (PPE), which includes N95 respirators and disposable gowns and gloves for standard airborne and contact precautions.^12^ Patients should also wear a mask during all encounters, especially because they can be contagious during the disease’s incubation period.

A high index of suspicion for ACS should be maintained for any patient presenting with symptoms of angina or angina equivalent. In addition to a good history and physical examination, the treatment team should evaluate patients using point-of-care ultrasound examination, electrocardiogram (EKG), and cardiac biomarkers.^13^ Simultaneous assessment of potential COVID-19 infection should begin at the point of first contact because it can protect the care team and determine the strategy for managing ACS. Currently, hospitals are testing all admitted patients for COVID-19 to optimize care and protect healthcare workers.

It is important to note that rising cardiac biomarkers in the setting of COVID-19 infection may be due not only to acute plaque rupture or erosion leading to ACS but also to direct myocardial injury, such as myocarditis. An increase in troponin is 13 times more common in severe COVID-19 infection than in milder presentations and has prognostic value.^14^ The same association was observed with the increase in B-type natriuretic peptide.^15^ It is important to differentiate a type 1 MI from a supply-demand mismatch (eg, respiratory failure in the setting of underlying coronary artery disease, arrhythmias), immune- and inflammatory-mediated myocarditis, pulmonary embolism, or stress-induced cardiomyopathy in the setting of COVID-19 infection.^16^ A case series from New York City in the beginning of the pandemic showed that, out of 18 patients infected with COVID-19 who developed ST-segment elevations on the EKG, 10 patients were found to have noncoronary myocardial injury.^17^ Subsequently, in a large registry of patients diagnosed with COVID-19 infection and presenting with typical or atypical symptoms consistent with ACS, and an EKG finding of ST-segment elevation, no culprit vessel lesion was found on angiography in 23% of patients (41 of 179); this was significantly higher than a finding of 1% of patients (5 of 459) in a similar matched control group from the pre–COVID-19 era.^18^ A history, troponin trend, serial EKGs, and other noninvasive imaging modalities can be helpful to establish a diagnosis. Point-of-care ultrasound is valuable in this population to help differentiate a STEMI from STEMI “mimickers,” which are more commonly encountered in patients with COVID-19 pneumonia. The administration of thrombolytic agents in the latter population could be catastrophic and cause significant harm to such patients.^19^

## Medical Management

Once ACS is suspected, medical therapy should be instituted immediately along with a decision whether to proceed with an invasive strategy. Medical therapy for ACS in COVID-19 patients is identical to patients without COVID-19. This includes dual antiplatelet therapy (aspirin and a P2Y12 inhibitor), intravenous or subcutaneous anticoagulation, statins, and beta blockers (if no contraindications). Oxygen is only administered in the setting of hypoxia. Reports from the early pandemic phase showed that angiotensin converting enzyme inhibitors and angiotensin-receptor blockers might be harmful, but further investigations negated these findings.^20,21^ In addition, a recent randomized controlled trial confirmed that there was no difference in short-term outcomes for patients with mild-to-moderate COVID-19 symptoms, regardless of whether such medications were continued or discontinued.^22^

## ST-Elevation Myocardial Infarction

There is strong evidence supporting the superiority of PPCI over fibrinolysis for reducing short-term complications and mortality in patients presenting with STEMI.^23^ PPCI is always preferred over fibrinolysis whenever adequate PCI facilities are available, and this should continue to remain the standard of care in patients presenting with STEMI with a low suspicion of COVID-19. Because there is a significant mortality benefit in patients receiving PPCI over intensive pharmacological therapies, especially in cases of anterior wall MI and concurrent cardiogenic shock, PPCI should still be the preferred approach for patients with high suspicion of COVID-19 presenting with cardiogenic shock and anterior STEMI.

Providing necessary PPE and training the catheterization laboratory staff in its correct use could decrease the spread of infection during the procedure. Moreover, using dedicated rooms for patients with known or presumptive positive COVID-19 infection and limiting the number of healthcare workers to only those who are essential for procedure support can further help control in-hospital spread of infections.^24^

It is important to note that COVID-19 patients are hypercoagulable; therefore, the healthcare team must pay particular attention to maintaining activated clotting time in the target range while performing PPCI. In a single-center study including 115 consecutive STEMI patients undergoing PCI, COVID-STEMI patients had higher rates of multivessel thrombosis with higher need for aspiration thrombectomy and glycoprotein IIb/IIIa use, higher inpatient mortality, greater rates of ICU admission, and longer length of stay compared with non-COVID STEMI patients treated during the same period.^25^

The North American COVID-19 Myocardial Infarction (NACMI) registry was created by the SCAI, American College of Cardiology, and Canadian Association of Interventional Cardiologists to fill the knowledge gap regarding outcomes in patients with diagnosed COVID-19 presenting with STEMI. Results from this prospective observational registry from 64 US and Canadian sites showed that only 78% of the patients with diagnosed COVID-19 infection who presented with STEMI received an angiogram compared to > 99% in the control group, and a higher number of patients received medical therapy (20% vs 2%).^18^ Furthermore, patients with COVID-19 had higher average door-to-balloon times (79 minutes vs 66 minutes; *P* = .008). This was associated with higher rates of in-hospital deaths, recurrent MIs, and repeat unplanned revascularization in the group diagnosed with COVID-19 compared with controls (36% vs 13%; *P* ≤ .001).^18^

Before COVID-19 vaccines became available, there were concerns regarding balancing risk of staff exposure during PPCI in a COVID-19–positive patient and risk of mortality and morbidity from a STEMI patient treated conservatively or with fibrinolytics alone. A decision-analysis framework was performed to evaluate this tradeoff.^10^ Using the best available data to estimate risk of mortality from STEMI and staff infection risk at that time, the authors found that for the majority of patients, PPCI offers mortality benefit over fibrinolysis. However, in those with severe COVID-19 and nonanterior STEMI without the presence of cardiogenic shock, the mortality benefit of PPCI was minimal and fibrinolytic therapy could be considered to reduce provider risk of COVID-19 infection. Given the highly efficacious and safe vaccines against COVID-19, this important ethical question and its implications may be significantly diminished, especially in health systems where all staff wear PPE and PPCI remains the standard of care for STEMI even in patients with COVID-19.

Fibrinolysis in patients presenting with STEMI in the setting of pneumonia has been independently associated with increased in-hospital mortality, especially among patients aged > 60 years.^26^ Another concern with fibrinolysis is risk of bleeding in STEMI mimickers, such as myocarditis. Hence, PPCI with adequate precautions remains the standard of care in STEMI patients with COVID-19. A fibrinolysis-based strategy may be considered at non-PCI capable referral hospitals or in specific situations where primary PCI is deemed inappropriate.

## Non-st Elevation Myocardial Infarction

For patients presenting with symptoms concerning for ACS with elevated troponin and no ST-segment elevation, COVID-19 screening with highly accurate reverse transcription polymerase chain reaction should be performed as soon as possible. Risk can be assessed using traditional scoring systems, such as Thrombolysis in Myocardial Infarction (TIMI) and Global Registry of Acute Coronary Events (GRACE) scores, and serial EKGs, troponin, and clinical monitoring should be performed. If there are no urgent indications for revascularization, PCI can be deferred until the patient’s infectivity status is ascertained.

## Care After Myocardial Infarction

Hospitalization remains one of the risk factors for COVID-19 infection.^27^ Thus, efforts should be made to reduce the hospital stay whenever possible. A common practice after PCI involves admitting all patients to critical care units for close observation, which frequently leads to a longer hospital course.^28^ To reduce length of hospital stay, the patient’s risk of contracting in-hospital COVID-19 infection, and utilization of critical care beds and resources, it is helpful to stratify risk for complications after STEMI revascularization using validated risk-predicting scores, such as CADILLAC or Zwolle risk scores, and triage patients based on risk of post-STEMI complications.^29,30^ The pandemic has made it necessary to increase emphasis on the discharge planning process to prevent recurrent ACS and avoid hospital readmission, thereby reducing the risk of in-hospital COVID-19 infection.^31^ Close follow-up with an outpatient cardiologist, either in office or by telemedicine, should be arranged before discharge.

## Current and Future Perspectives

COVID-19 vaccinations have shown to be safe and effective in preventing cases and reducing the severity of the disease, hospitalization, and death.^32^ However, there is significant vaccination hesitancy recorded in different parts of the country, and this—along with new emerging strains like the Delta, Delta-plus, Omicron, and Lambda variants—continues to pose risks for a wave of milder COVID-19 infections in the near future.^33^ The factors that resulted in increased complications and mortality from ACS in past waves will continue to persist in future waves of milder disease. It is important for healthcare systems to evolve and create policies to ensure that treatment is not delayed and standard of care is maintained for patients presenting with ACS in future similar circumstances. The use of telemedicine played a major role in providing healthcare services during the pandemic.^34^ The effectiveness of telemedicine for preventive cardiology and post-ACS care should be studied further since it could result in regular healthcare follow-up without visiting a cardiologist’s office.

## Key Points

- The COVID-19 pandemic has directly and indirectly impacted the care of patients with cardiovascular emergencies. COVID-19–positive patients with acute coronary syndrome (ACS) present late and have higher incidences of cardiogenic shock and in-hospital mortality compared with the pre–COVID-19 ACS population.
- Primary percutaneous coronary intervention with full personal protective equipment and adequate protection in the catheterization lab remains the mainstay for patients presenting with STEMI and confirmed or a high index of suspicion for COVID-19 infection.
- Efforts are needed to increase public health messaging and education regarding avoiding delays and seeking immediate medical care for suspected acute cardiac conditions and to increase vaccination against COVID-19, which has been proven to be safe and effective.
